# Addenbrooke's Hospital, Cambridge

**Published:** 1917-08-25

**Authors:** 


### ADDENBROOKE'S HOSPITAL, CAMBRIDGE.

A General Quarterly Court of the President and Governors was held at the hospital on August 13. The General Committee reported that the expenditure for the quarter was £4,624, against £3,895 in the corresponding quarter of last year, the increase being accounted for by the continual advance in the price of all commodities and by an increase of sixty-five in the daily average number of civil patients and wounded soldiers admitted during the quarter.

The Committee reported with great pleasure and gratitude the receipt of £1,000 from Mr. Almeric Paget towards the maintenance of two cots for children, to be named after the late Mrs. Almeric Paget and Mr. Almeric Paget respectively. Mr. and Mrs. Almeric Paget had previously given a handsome contribution to the building fund, and the late Mrs. Paget most kindly gave a contribution of £750 towards the cost of providing the electric lift, which has proved a great boon to the hospital.

It is hoped that the negotiations with the County Council of Cambridgeshire with reference to the establishing of a clinic at the hospital for dealing with cases of venereal disease will be completed very shortly.

Sir Clifford Allbutt's Great Services.

The Committee acknowledge with gratitude the great services to the hospital of Sir Clifford Allbutt, Regius Professor of Physic, and of Dr. F. Shillington Scales at this difficult time. Sir Clifford Allbutt has been acting as honorary physician in the absence of Dr. Laurence Humphry, and Mr. Brown and Dr. Shillington Scales have been taking the place of Mr. Arthur Cooke in the out-patient department. Dr. Shillington Scales has given in addition a very large amount of time to other work of the hospital. In this connection tribute is paid to the services of Dr. Aldren Wright, who, in addition to fulfilling the duties of honorary physician to the hospital, has, as secretary of the honorary medical and surgical staff, by his energy and tact succeeded in maintaining the medical and surgical work of the hospital in a state of continuous efficiency. The best thanks of the Governors was given to the whole of the medical and surgical staff, and to the members of the nursing and clerical staff, for the efficient and zealous way in which, under great difficulties, they had carried on the work of the hospital.

Gifts to the Hospital.

£2,000, free of duty, to the endowment fund of the hospital, from the late Mr. T. Brooks Bumpsted, of Leighton, Trumpington Road, Cambridge. The residue of his property, real and personal, subject to a life interest, for the general use and purposes of the institution, from the late Mr. Joseph Turner Bailey, of Cambridge. This bequest consists largely of freehold property producing an income of about £13 to £15 per week. £100 from the executors of the will of Miss Elizabeth Esther Bridges, deceased, late of Cambridge, being the amount bequeathed to the hospital in 1894, payable on the death of the survivor of three annuitants named in the will, the decease of the said surviving annuitant having taken place in May 1917.

